# Probiotic-Based Cleaning Solutions: From Research Hypothesis to Infection Control Applications

**DOI:** 10.3390/biology14081043

**Published:** 2025-08-13

**Authors:** Matthew E. Falagas, Dimitrios S. Kontogiannis, Maria Sargianou, Evanthia M. Falaga, Maria Chatzimichali, Charalambos Michaeloudes

**Affiliations:** 1 Alfa Institute of Biomedical Sciences (AIBS), 9 Neapoleos Street, Marousi, 151 23 Athens, Greece; d.kontogiannis@aibs.gr (D.S.K.);; 2 School of Medicine, European University Cyprus, Nicosia 2404, Cyprus; c.michaeloudes@euc.ac.cy; 3 Department of Medicine, Tufts University School of Medicine, Boston, MA 02111, USA; 4 Department of Medicine, Hygeia Hospital, 151 23 Athens, Greece

**Keywords:** antimicrobial resistance, antimicrobial resistance gene, *Bacillus*, detergent, disinfectant, healthcare-associated infection, infection control, *Lactobacillus*, probiotic-based cleaning solution

## Abstract

Novel infection control practices are necessary to reduce the incidence of healthcare-associated infections (HAIs). Since 2007, probiotic-based cleaning solutions have been proposed as an alternative to traditional methods using disinfectants and detergents in healthcare settings, including hospitals. We conducted a comprehensive search across Google Scholar, PubMed, Scopus, and Web of Science resources. Studies that assessed the reduction in pathogens on surfaces and the emergence of HAIs after the use of probiotic-based cleaning solutions were eligible for evaluation. A total of 16 studies (13 in clinical settings and 3 on experimental surfaces) were included. The *Staphylococcus* species were most commonly identified before and after the use of probiotic-based cleaning solutions. All studies showed numerically lower pathogen counts and fewer HAIs after using probiotic-based cleaning solutions compared to disinfectants and detergents. Three studies indicated a reduction in antimicrobial resistance genes after use of probiotic-based cleaning solutions. One of these showed statistically significant differences compared to traditional disinfectants (alcohol, amines, and quaternary ammonium compounds) and detergents (non-ionic and anionic surfactants). The results of the included studies suggest the consideration of probiotic-based cleaning solutions for infection control in healthcare systems. However, given the novelty of this approach, further studies are needed to verify the evaluated findings and investigate the short- and long-term effectiveness, and safety of probiotic-based cleaning solutions on infection control practices in healthcare settings.

## Simple Summary

Healthcare-associated infections are a significant global problem. Besides other interventions, including the appropriate use of antimicrobial agents, infection control practices are essential to reduce the incidence of healthcare-associated infections. In December 2007, one of the authors of this article (Professor Matthew Falagas) made a research hypothesis regarding the potential usefulness of the application of probiotics on surfaces in healthcare settings for the control of healthcare-associated infections. At that time, microbiological evidence suggested that probiotics may antagonize pathogens on inanimate surfaces. Since then, there have been efforts for the application of probiotic-based cleaning solutions in infection control practice. In this article, we evaluated clinical and experimental evidence regarding sanitation using probiotics compared to other products used, such as detergents and disinfectants. The emerging data are encouraging, as probiotics reduce the pathogen counts on environmental healthcare setting surfaces. These data suggest that probiotic-based cleaning solutions may be considered in healthcare settings. However, more studies are needed to investigate the effectiveness and safety of probiotics for cleaning purposes in healthcare settings. Additionally, further development of probiotic-based cleaning solutions should be standardized based on the strict guidelines of the relevant regulatory agencies, similar to those for disinfectants, to ensure consistency and reliability.

## 1. Introduction

Antimicrobial resistance has become a top global public health priority, and is associated with significant morbidity, mortality, and costs [1]. Healthcare-associated infections (HAIs) are frequently caused by pathogens that are resistant to multiple antibiotic classes, rendering them multidrug-resistant (MDR), extensively drug-resistant (XDR), and even pan-drug-resistant (PDR) [2,3]. There are limited therapeutic options, if any, for such infections, and thus, they are associated with considerable mortality [4,5]. These facts necessitate the exploration of novel infection control practices to mitigate the frequency and impact of HAIs [6,7].

In December of 2007, one of the authors of this work (M.E.F.) submitted an article analyzing a research hypothesis on the potential application of probiotics and biosurfactants for controlling nosocomial infections (the article was published in 2009) [8]. At that time, preliminary environmental microbiological evidence suggested that probiotics may antagonize nosocomial pathogens on inanimate surfaces, just as they do in the human body, as part of the microbiome [8]. The growth of probiotics on inanimate surfaces in healthcare units, including hospitals, may lead to a reduction in the number of pathogens, and this may result in a decrease in HAIs [8].

Additionally, the COVID-19 pandemic led to the extensive use of antiseptics, raising concerns about their safety, including skin reactions and exacerbation of chronic obstructive pulmonary disease (COPD) [9,10]. Consequently, there has been an increased need for the development of alternative infection control practices. Probiotic-based sanitation solutions have garnered the attention of several infection control practitioners.

Probiotic-based cleaning solutions are cleaning products that comprise live microorganisms that are spore-forming, such as *Bacillus* species pluralis (spp.) (most commonly *Bacillus subtilis*, *Bacillus megaterium*, and *Bacillus pumilus*) [11]. These solutions are delivered in powder format, liquid concentrate format (usually diluted with water in spray bottles), or in ready-to-use liquid format (pre-diluted solution). For the maintenance of these solutions, *Bacillus* spp. bacteria are preserved in their spore form that can withstand extreme environmental conditions, such as high temperatures. The products are sometimes stored in opaque containers that protect them from ultraviolet (UV) radiation.

The cleaning products used nowadays in healthcare settings are disinfectants that are composed mainly of alcohol, chlorine compounds, quaternary ammonium compounds, phenols, or aldehydes [12]. Disinfectants are delivered in liquid, foam, and gel formats, as wipes, or as aerosol sprays. Additionally, detergents are used and mainly composed of surfactants that are divided into cationic, anionic, non-ionic, and amphoteric types, and reduce surface tension [13]. Detergents are delivered in liquid or powder format.

In this context, we evaluated the evolving literature from the publication of the research hypothesis article to the applications of probiotic-based cleaning solutions in infection control. We focused on their effect on measurable outcomes, including the growth of bacteria on environmental, inanimate hospital surfaces, HAIs, and antimicrobial resistance. Our article addresses both clinical and experimental evidence regarding probiotic-based sanitation since the original hypothesis proposed in 2007.

## 2. Methods

### 2.1. Eligibility Criteria

Studies were included in this study if they used probiotic-based cleaning solutions to clean surfaces in clinical or laboratory settings. All studies that compared the use of probiotic-based cleaning solutions with other cleaning solutions (disinfectants or detergents) were included. Comparative studies that used each of these cleaning solutions (disinfectants, detergents, or probiotic-based cleaning solutions) on different surfaces (in clinical wards or rooms) during the same period were included. Also, pre-post interventional studies that applied each solution consecutively after a specific period of time on the same surface were included. Studies that assessed the use of probiotic-based cleaning solutions in non-clinical surfaces (in laboratories) were also included. There was no restriction on the language of publication, journal type, or region of study. Studies that evaluated the use of probiotic-based cleaning solutions with phages, as well as conference abstracts, were excluded from further evaluation.

### 2.2. Identification of Relevant Studies and Search Strategy

Four resources (Google Scholar, PubMed, Scopus, and Web of Science) were utilized to identify relevant articles. A search strategy using the keywords “probiotic”, “sanitation”, and “cleaning” was implemented on 25 May 2025 for PubMed, Scopus, and Web of Science, and on 3 June 2025 for Google Scholar (Supplementary Table S1). The studies were first screened via title and/or abstract, and then by full text.

### 2.3. Data Extraction and Tabulation

The extracted and tabulated data included the presence of pathogens and *Bacillus* spp. isolates on the surfaces before and after the intervention (use of probiotic-based cleaning solutions). The presence of isolates was assessed in terms of colony forming units (CFUs) per square meter of surface (CFU/m^2^). Additionally, the effect of probiotic-based cleaning solutions on antimicrobial resistance genes and the emergence of HAIs compared to other traditional solutions (disinfectants and detergents) were evaluated.

### 2.4. Outcomes of the Included Studies

Specifically, the following outcomes were evaluated: the effect of probiotic-based cleaning solutions on (a) the number of pathogens (in CFU/m^2^ or CFU, depending on the data provided by each study) on the tested surfaces, (b) the number of *Bacillus* strains (in CFU/m^2^ or CFU), (c) the transfer of resistance genes between pathogens and/or *Bacillus* strains, and (d) the emergence of HAIs of patients in clinics where probiotic-based cleaning solutions were applied. Additionally, the costs associated with HAIs and antimicrobial drug usage, as well as material expenses, were analyzed. Also, the antimicrobial resistance of pathogens to various clinically used antibiotics was evaluated after using probiotic-based cleaning solutions.

## 3. Results

### 3.1. Identification of Relevant Studies

**Figure 1:**
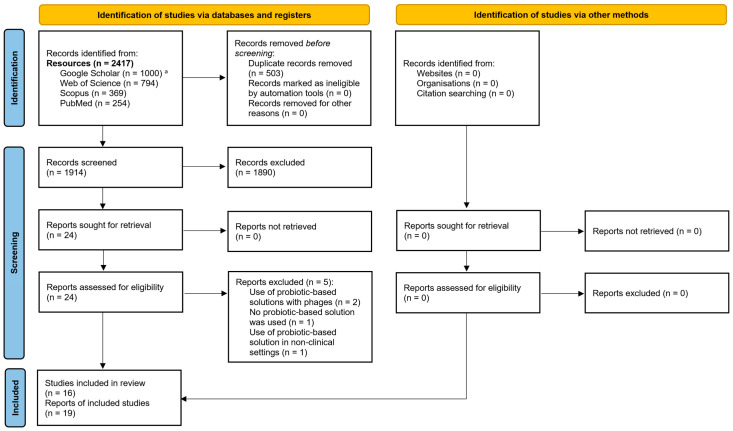
“Preferred Reporting Items for Systematic Reviews and Meta-Analyses” (PRISMA) flow diagram for the identification, screening, and selection of articles. Notes: ^a^ For Google Scholar, out of 39,700 results, only the first 1000 articles could be accessed. https://www.bmj.com/content/372/bmj.n71 (accessed on 17 June 2025).

Figure 1 presents the “Preferred Reporting Items for Systematic Reviews and Meta-Analyses” (PRISMA) flow diagram for identifying, screening, and selecting relevant articles. A total of 2417 articles were identified, and after deduplication, 1914 articles were screened based on their title and/or abstract. Finally, 24 articles were evaluated by reading the full text; 5 were excluded, and 19 articles were eligible for inclusion [11,14,15,16,17,18,19,20,21,22,23,24,25,26,27,28,29,30,31]. Six articles reported different aspects of three single studies (two articles for each study) [16,17,18,19,25,26]. Thus, a total of 16 studies were included in this article.

### 3.2. Results of the Included Studies

Table 1 presents the characteristics of the included studies that assessed the use of probiotic-based cleaning solutions in clinical settings. In total, six prospective, comparative, interventional studies [14,15,16,18,22,24,30], five pre–post interventional studies [17,19,20,28,29,31], one prospective, non-randomized controlled trial [25,26], and one cluster-randomized, controlled, crossover trial [27] were included. All studies were conducted in hospital wards, except for one study that took place in dental clinics [15]. Probiotic-based cleaning solutions contained various *Bacillus* strains, most frequently *Bacillus megaterium*, *Bacillus pumilus*, and *Bacillus subtilis*. The probiotic-based cleaning solutions were applied for several months, with durations varying across studies and ranging from 1 to 6 months. The controlled solutions used in each study varied, including detergents and disinfectants. The products used in the included studies contained chlorine, alcohol, quaternary ammonium compounds, or non-ionic and anionic surfactants.

Table 2 presents the outcomes after the application of probiotic-based cleaning solutions for surfaces in clinical settings, as described above. Before the application of probiotic-based cleaning solutions, the most abundant pathogens on the tested surfaces were *Staphylococcus* spp., as indicated by studies that provided relevant data [18,20,22,24]. Specifically, in a prospective, interventional, comparative analysis, 96.5% of the total median pathogen load of 22,737 CFU/m^2^ (range, 17,053–60,632 CFU/m^2^) was attributed to *Staphylococcus* spp., with a median pathogen load of 21,895 CFU/m^2^ (range, 13,684–57,263 CFU/m^2^) [18]. Other less frequent pathogens were also observed, such as Enterobacterales, *Acinetobacter* spp., *Pseudomonas* spp., and *Clostridium difficile* (*C. difficile*) [18]. In another prospective interventional study, *Staphylococcus aureus* (*S. aureus*) was the most abundant species identified with a load of 400 CFU/m^2^ compared to 250, 200, and 50 CFU/m^2^ for *Enterococcus faecalis* (*E. faecalis*), *Pseudomonas aeruginosa* (*P. aeruginosa*), and *Candida albicans* (*C. albicans*), respectively [22].

In a pre–post interventional study, the *Staphylococcus* spp. were also the most abundant species with a median load of 6316 CFU/m^2^ in rooms (of a total median 7158 CFU/m^2^) and 17,053 CFU/m^2^ in bathrooms (of a total median 20,211 CFU/m^2^), accounting for 88% of the total pathogen load in the first study hospital. A median load of 7921 CFU/m^2^ in rooms (of a total median 8790 CFU/m^2^) and 14,948 CFU/m^2^ in bathrooms (of a total median 16,420 CFU/m^2^), accounting for 91% of the total pathogen load in the second study hospital [20].

Two prospective, interventional, comparative studies provided relevant data on the isolated pathogens after the use of probiotic-based cleaning solutions [15,16]. In one study, it was shown that more than 50% of isolates were coagulase-negative staphylococci after 3 weeks of daily cleaning with probiotic-based cleaning solutions [15]. Other pathogens were also reported, such as *Staphylococcus saprophyticus* (four isolates), *Staphylococcus hemolyticus* (two isolates), *Staphylococcus hominis* (1 isolate), *Staphylococcus simulans* (1 isolate), and *Staphylococcus epidermidis* (one isolate) [15]. In another study, it was shown that *Staphylococcus* spp. were the most abundant pathogens, with a mean of 4674 CFU/m^2^ compared to other pathogens, such as *Candida* spp. (1108 CFU/m^2^), *Acinetobacter baumannii* (*A. baumannii*) (520 CFU/m^2^), *P. aeruginosa* (415 CFU/m^2^), Enterobacterales (189 CFU/m^2^), *C. difficile* (132 CFU/m^2^), and *Aspergillus* spp. (12 CFU/m^2^) [16].

In a pre–post interventional study, the effect of probiotic-based cleaning solutions on the population of the fungi *Candida*, *Aspergillus*, and *Fusarium* was evaluated using qualitative polymerase chain reaction (PCR) [19]. It was shown that after 6 months of probiotic-based cleaning solutions use, the *Candida* spp. load decreased from 6500 genomes/100 m^2^ to 0.25 genomes/100 m^2^, the *Aspergillus* spp. load decreased from 40 genomes/100 m^2^ to 2.6 genomes/100 m^2^, and the *Fusarium* spp. load remained unaffected at 5 genomes/100 m^2^ [19].

A pre–post interventional study demonstrated that the use of probiotic-based cleaning solutions led to an increase in *Bacillus* strains from a mean (± standard deviation [SD]) of 6.7 ± 3.1% to 66.0 ± 5.5% after 4 months [17]. Among the 6 out of 159 (3.8%) patients who developed an HAI, blood and urine samples were collected, and were negative for the detection of *Bacillus* strains using PCR [17].

A prospective, multicenter, pre–post interventional study demonstrated that the use of probiotic-based cleaning solutions resulted in statistically significantly fewer HAIs compared to the control, which consisted of typical cleaning solutions with chlorine [17,18]. More specifically, urinary tract infections (UTIs) were the most common HAI in the probiotic-based cleaning solutions (70/141 [50%]) and control (179/314 [57%]) groups [17,18]. Next was bloodstream infection (BSI) in the probiotic-based cleaning solutions (31/141 [22%]) and control (54/314 [17%]) groups [17,18]. Others, such as gastrointestinal, skin and soft tissue, lower respiratory tract, eye, ear, nose, and throat infections, were also reported in both groups in smaller percentages (ranging from 1.4 to 5.7% in the probiotic-based cleaning solutions and from 0.3 to 7% in the control) [17,18]. One patient developed a reproductive tract infection in the control group (1/314 [0.3%]). Additionally, in the probiotic-based cleaning solutions group, one patient developed a bone and joint infection, and another an intra-abdominal infection (for each 1/141 [0.7%]) [17,18].

The pathogen most commonly isolated from patients with HAI was *Escherichia coli* (*E. coli*), found in both the probiotic-based cleaning solutions (27/137 [19.7%]) and the control groups (93/332 [28%]) [17,18]. The next most common pathogen was *Enterococcus* in cases using probiotic-based cleaning solutions (24/137 [17.5%]) and control groups (57/332 [17.2%]). Other pathogens isolated from patients with HAI included *S. aureus*, *Staphylococcus* spp., *Streptococcus* spp., *Klebsiella* spp., *P. aeruginosa*, *Proteus mirabilis*, *C. difficile*, *Enterobacter* spp., *A. baumannii*, and *Candida* spp. In addition, infections due to viruses developed in the probiotic-based cleaning solutions (3/137 [2.1%]) and control (12/332 [3.6%]) groups [17,18]. After the use of probiotic-based cleaning solutions, no isolation of *Citrobacter* spp., *Morganella* spp., or other Enterobacterales was observed from patients with HAIs [17,18]. In contrast, these pathogens were isolated from patients with HAIs after the use of traditional sanitation methods in the control group (3/332 [0.9%] for each *Citrobacter* spp. and *Morganella* spp., and 1/332 [0.3%] for other Enterobacterales) [17,18].

However, *Bacillus* spp. caused no HAIs, and the probiotic-based cleaning solutions were a significantly independent protective factor for the emergence of HAIs (OR [95% CI]: 0.44 [0.35–0.54], *p* < 0.0001) [17,18]. In a multivariate analysis, it was demonstrated that probiotic-based cleaning solutions were a protective factor against the acquisition of HAI (OR 0.44, 95% CI [0.35–0.54], *p* < 0.001) [17,18]. In contrast, the length of stay (OR 1.08, 95% CI [1.07–1.09], *p* < 0.001) and the use of urinary catheter (OR 2.68, 95% CI [2.10–3.41], *p* < 0.001) were significantly associated with HAI onset [17,18].

In another pre–post interventional study, the severity of HAIs was assessed using the Australian Incident Monitoring System (AIMS) [29]. The results showed that moderate-to-severe HAIs were observed in 111/203 (54.6%) cases using conventional cleaning and in 46/106 (43.4%) cases using probiotic-based cleaning solutions, respectively [29]. Severe outcomes, such as severe disability or death, were reported in 3/200 (1.5%) cases using conventional cleaning and in 1/111 (0.9%) cases using probiotic-based cleaning solutions [29].

Considering antimicrobial resistance, all resistance genes tested (including metallo-β-lactamases [MBLs] and extended-spectrum β-lactamases [ESBLs]), except for the “*msrA*” gene, showed a decrease after the use of probiotic-based cleaning solutions compared to the control group (before the use of probiotic-based cleaning solutions) [17]. The “*S. aureus* identification gene” and the “*SpA*” genes exhibited a more than 3-log decrease compared to the control group [17]. In another study, the “*S. aureus* identification gene” showed the most considerable reduction after using probiotic-based cleaning solutions compared to the other antimicrobial genes, with a similar reduction (99.9%) [16].

Some studies showed that the cost of antimicrobial treatment due to HAIs, as well as the material costs, were numerically lower for patients in areas where probiotic-based cleaning solutions were used, compared to disinfectants or detergents [16,26,29]. A prospective, comparative, interventional study showed that the management costs of each HAI were a mean (±SD) of EUR 116.3 ± 249.9 after using probiotic-based cleaning solutions compared to a mean (±SD) of EUR 213.7 ± 915.3 after conventional cleaning with chlorine-containing products [16]. A pre-post interventional study showed that the cost of antimicrobial treatment for each patient with at least one HAI was EUR 110 after using probiotic-based cleaning solutions and EUR 272 after using a conventional cleaning solution [29]. In another study, it was found that the weekly material costs for the probiotic-based cleaning solutions were ZAR 2844.90, compared to ZAR 2885.34 for the control solutions used (liquid soap detergent, pine liquid disinfectant, and ammonia) [26].

Table 3 presents the characteristics and outcomes of the studies using probiotic-based cleaning solutions in experimental (non-clinical) surfaces. One study used the “UNI EN 14476:2019” standard procedure [32]. This study demonstrated that the use of probiotic-based cleaning solutions resulted in a virus load reduction of all tested viruses, regardless of the probiotic-based cleaning solutions dilution (1:10, 1:50, or 1:100), including herpes simplex virus 1 (HSV-1), modified vaccinia virus Ankara (MVA), and severe acute respiratory syndrome coronavirus 2 (SARS-CoV-2) [21]. In a simulation study, the inclusion of probiotic-based cleaning solutions resulted in a reduction in the number of *A. baumannii* and *K. pneumoniae* isolates [23]. The surfaces were gradually dominated by *Bacillus* strains [23]. In another study, the survival of *S. aureus* and *E. coli* was limited after the use of probiotic-based cleaning solutions [11].

Table 4 presents the proportions of pathogens’ resistance to various antimicrobial agents after using probiotic-based cleaning solutions and controls, respectively, in the two studies that provided relevant data [15,16]. No statistically significant differences were found between the two groups in terms of the tested antibiotics, except for cefepime, where it was demonstrated that the use of probiotic-based cleaning solutions resulted in a significant decrease in the resistance of Gram-negative rods to this antibiotic compared to the control group [15].

## 4. Discussion

A notable body of evidence suggests the consideration of probiotic-based cleaning solutions in infection control applications within modern healthcare settings. According to the results of the included studies, surfaces in healthcare facilities are often contaminated with numerous bacteria, notably *Staphylococcus* spp. However, the application of probiotic-based cleaning solutions resulted in a larger decrease in the total number of pathogens in all studies compared to other cleaning methods (disinfectants and detergents). Although there were no statistically significant differences, except for one study that favored probiotic-based cleaning solutions, the evaluation of the published evidence suggests that these solutions are at least as effective as traditional disinfectants and detergents.

The biological mechanism of the effect of probiotic-based cleaning solutions is bacterial interference, i.e., the ability of non-pathogenic bacteria, such as *Bacillus* spp., to inhibit or outcompete pathogenic bacteria. Bacterial interference is the underlying mechanism of activity of probiotics in multiple applications in modern clinical practice, including vulvovaginal candidiasis and bacterial vaginosis [33,34,35]. According to the included studies, bacterial interference has been demonstrated through various mechanisms using probiotic-based cleaning solutions on surfaces. Specifically, probiotic-based cleaning solutions reduce the pathogen load on surfaces, decrease the number of antimicrobial resistance genes, and inhibit the formation and adherence of biofilms [36]. The main mechanisms through which probiotic-based cleaning solutions exert bacterial interference are competition for surface colonization and nutrient acquisition, as well as the release of antimicrobial metabolites [19]. These metabolites include bacteriocins, which disrupt the bacterial cell membrane and cause DNA damage; biosurfactants that cause cell membrane disintegration and inhibit surface adhesion; and organic acids, which impair bacterial growth by lowering environmental pH [37]. Additionally, probiotics may inhibit biofilm formation by disrupting quorum sensing through the release of specific metabolites [38].

Further studies are needed for addressing the safety concerns associated with probiotic-based cleaning solutions [39]. Probiotics are live microorganisms and, therefore, may cause opportunistic infections in patients with severely impaired immune systems, including those who have undergone bone marrow or solid organ transplants, as well as patients with various forms of neoplasia under immunosuppressive therapy [40,41,42]. More well-designed and conducted studies are needed to ensure the safety of probiotic-based cleaning solutions. Such studies should also examine the potential toxicity associated with the inhalation of probiotics included in probiotic-based cleaning solutions and their impact on the antimicrobial resistance of the microbiome in healthcare facilities [19,43]. Although no research has documented bloodstream infections caused by *Bacillus* strains from cleaning solutions, additional long-term studies are necessary to investigate the long-term effects and safety of chronic exposure to these solutions in healthcare settings.

In general, the quality and quantity of scientific studies on probiotics are lower than those of antibiotics and other fields related to infectious diseases and microbiology. This has been demonstrated in several articles on the effectiveness of probiotics in preventing and treating various infections, including upper and lower respiratory infections, ventilator-associated pneumonia, urinary tract infections, and abdominal surgical infections [44,45,46,47,48]. Similar conclusions were made in articles on the use of probiotics in allergic rhinitis, asthma, and atopic dermatitis [49,50].

Regulatory agencies such as the World Health Organization (WHO), the European Centre for Disease Prevention and Control (ECDC), and the Centers for Disease Control and Prevention (CDC) have published structured guidelines regarding infection control programs at the national level, the European Union, and the United States [51,52,53]. These agencies should continue their efforts to also improve the standardization of probiotic-based cleaning solutions, including their composition (specific species and quantities). In this direction, the recently published guidelines of the European Union Council offer significant input [54]. The efforts to improve regulatory standardization also apply to the various formulations of probiotics available for use in food or as prevention or treatment options in clinical practice. The probiotic-based cleaning solutions are not regulated as pharmaceutical agents, although there are efforts in several countries to improve their regulatory status, including Germany. Specifically, in Europe, there is no regulatory approval by the European Chemicals Agency (ECHA) based on the Biocidal Products Regulation (BPR) for hospital use of probiotic-based cleaning solutions. In countries such as Italy, Belgium, and Germany, these solutions have been used as part of research studies. Also, the United States Environmental Protection Agency has not yet registered probiotic-based cleaning solutions. Instead, the probiotic-based cleaning solutions are regulated as consumer products, and thus their safety and standardization process is not as thorough as those for drugs [55].

Comparing probiotic-based cleaning solutions with traditional disinfectants and detergents for use in healthcare facilities reveals the distinct advantages and disadvantages of this new approach to infection control. Probiotic-based cleaning solutions have a positive effect on the microbiome of healthcare facilities and thus have an ecological profile. Additionally, they may be associated with fewer adverse events on healthcare personnel and patients, including a lower probability for inhalation toxicity and eczema [43,56]. Additionally, no acquired resistant genes have been reported after the use of probiotic-based cleaning solutions, at least by now [19]. Furthermore, a study demonstrated that the tested *Bacillus* spp. lacked virulence genes [57]. Another potential advantage of using probiotic-based cleaning solutions is that they may be cost-effective, based on the limited available data. Studies have shown a numerically lower cost of using probiotic-based cleaning solutions compared to other cleaning products (disinfectants and detergents) [16,26,29].

Probiotics can modulate resistome dynamics and horizontal gene transfer. In more detail, it was shown in an in vitro study that probiotics reduce the levels of antimicrobial resistance genes (ARGs), such as the *tetM*, *tetO*, and *bla*_TEM_ genes [58]. Also, in another study, it was shown that probiotics reduced the level of ARGs in individuals who were permissive to probiotic colonization [59]. Additionally, it was demonstrated that probiotics interfere with the transfer of ARGs and are active in quorum-sensing inhibition [60,61].

However, the use of probiotic-based cleaning solutions has some disadvantages. The activity of probiotic-based cleaning solutions is observed after several hours, compared to other cleaning products that have a more immediate effect [31]. Additionally, the combination of probiotic-based cleaning solutions and chemical solutions is ineffective because traditional disinfectants and detergents can negatively impact the probiotics included in the probiotic-based cleaning solutions [20]. Also, further studies are needed to demonstrate the effectiveness of probiotic-based cleaning solutions in reducing non-bacterial pathogens, including viruses and fungi [27,56]. Indeed, only one study reported the effects of probiotic-based cleaning solutions on viruses, and another one on fungi [19,21]. Additionally, the use of probiotic-based cleaning solutions is not recommended in sterile environments, such as operating rooms.

Also, a pragmatic study included in our analysis did not demonstrate the superiority of probiotic-based cleaning solutions over other cleaning solutions (disinfectant or soap-based solutions) in reducing the emergence of HAIs [27]. In other words, the positive effects of probiotic-based cleaning solutions, compared to traditional disinfectants and detergents, in reducing the bacterial load of pathogens from surfaces in healthcare settings were not directly translated into a reduction in HAIs [27]. Although this seems to be an unexpected finding, several reasons may contribute to this observation. First, the observed differences in the number of pathogens on surfaces in healthcare settings, using probiotic-based cleaning solutions compared to traditional disinfectants or detergents, were not statistically significant in the majority of studies. Second, even the reduced number of pathogens with probiotic-based cleaning solutions, if true, may be enough to initiate an HAI. Third, the transfer of pathogens between patients and from healthcare personnel to patients is a documented mechanism of cross-infection.

This study has limitations. Studies reporting the use of probiotic-based cleaning solutions in combination with phages were not included. However, some analyses have shown promising results, with a greater reduction in pathogen load compared to probiotic-based cleaning solutions alone [62,63]. Particularly, they have been reported to successfully counteract MDR species contaminating hospital environments, including *P. aeruginosa* and methicillin-resistant *S. aureus* [20]. Integrating bacteriophages with probiotic-based cleaning solutions is a promising strategy, as they exhibit rapid and species-specific lytic activity, potentially enhancing the delayed effects of probiotic interference alone. Thus, lytic phages spare human and probiotic cells, but adversely cause the release of endotoxins and inflammatory proteins from targeted pathogenic bacteria [62,64]. As a result, similar concerns to those associated with the yet unclear adverse effects of probiotic-based cleaning solutions in immunocompromised patients surround the use of phages in joint cleaning solutions, prompting further investigation into safety and clinical implementation.

Additionally, further studies will be necessary in clinical settings to determine the effect of probiotic-based cleaning solutions on the incidence of HAIs, the most relevant outcome. Although we did not perform a formal risk-of-bias assessment, the majority of studies were observational, characterized by heterogeneous designs and durations, thereby constraining the robustness of our conclusions, and precluding the synthesis of the available data using meta-analysis techniques. Finally, we did not include studies on the effectiveness and safety of probiotic-based cleaning solutions for surfaces beyond non-clinical or experimental settings, such as public transportation [65]. Future directions for the use of probiotic-based cleaning solutions include the conduction of large pragmatic studies to increase the robustness of the data regarding the effectiveness and safety of these solutions in healthcare settings. Implementing training programs and educating healthcare personnel on the proper maintenance and use of these solutions would also be important. Additionally, studies evaluating the environmental impact of the probiotic-based cleaning solutions, especially their effect on the microbial ecosystems, would be valuable before their widespread use in healthcare facilities. The cost effectiveness is also essential when implementing a new infection control practice in hospitals; thus, more studies assessing this aspect are needed. Finally, the development of probiotic-based cleaning solutions should be standardized based on strict regulatory agencies’ guidelines, similar to those for disinfectants, to ensure consistency and reliability.

## 5. Conclusions

The evaluation of the published evidence suggests that probiotic-based cleaning solutions are effective alternatives to traditional disinfectants and detergents in healthcare settings. Further studies are warranted to elucidate the comparative effectiveness, with a focus on the impact on HAIs, and the safety of probiotic-based cleaning solutions.

## Figures and Tables

**Table 1:** Characteristics of the included studies that used probiotic-based sanitation cleaning solutions for surfaces in clinical settings.

Author, Year [Ref #]	Period of Study, Country	Type of Study	Setting	Tested Surface	PBCS	Duration and/or Design of Probiotic Cleaning	Duration and/or Design of Control
Afinogrnova, 2018 [14]	NR, Russia	Prospective, comparative interventional	Two rooms in a medical center	Floor	NR	For 1 month	Disinfectants and detergents (not specified)
Al-Marzooq, 2018 [15]	2/2017–5/2017, United Arab Emirates	Prospective, comparative interventional	Three dental clinics	Dentist chair, drainage, handpiece wire, headrest, sides of patient chair, floor, keyboard, spittoon, and sink	*Bacillus subtilis*	For 3 weeks, daily	For 1 week, daily; floor: chemical solution (sodium lauryl ether sulfate and diethanolamide); other surfaces: disinfectant, (ethanol, 1-propanol, and quaternary ammonium compounds)
Caselli, 2016 [19]	NR, Italy	Pre-post interventional	A private hospital	Floor, bed, bathroom sink	*Bacillus megaterium*, *Bacillus pumilus*, *Bacillus subtilis* (10^7^ spores/mL, 1:100 dilution)	For 6 months	Pre-intervention: conventional cleaning with disinfectants
Caselli, 2016 [17]; 2018 [18]; 2019 [16]	1/2016–6/2016, Italy	Prospective, comparative interventional	Six hospitals (in 1 of 6 hospitals, only conventional cleaning was applied)	Bed, bed footboard, and sink	*Bacillus megaterium*, *Bacillus pumilus*, *Bacillus subtilis*	For 6 months, daily ^a^	Pre-intervention: for 6 months, conventional cleaning with disinfectants (chlorine-containing products)
D’Accolti, 2023 [20]	3/2021–6/2021, Italy	Pre–post interventional	Two hospitals (H1 and H2)	Room and bathroom surfaces	NR	For 1 month	Pre-intervention: conventional cleaning with chemical-based products
Klassert, 2022 [24]	NR, Germany	Prospective, comparative interventional	Neurology ward with nine rooms in a university hospital	Floor, door handle, and sink	*Bacillus amyloliquefaciens*, *Bacillus licheniformis*, *Bacillus megaterium*, *Bacillus pumilus*, *Bacillus subtilis*	For 3 months	Pre-intervention: disinfectants (alcohol, amines, and quaternary ammonium compounds) applied for 3 months, then detergents (non-ionic and anionic surfactants) applied for 3 months
Kleintjes, 2019 [25]; 2020 [26]	8/2017–9/2017, 2/2018 ^b^, South Africa	Prospective, non-randomized, controlled	A burn center (ward and ICU)	NR	*Bacillus megaterium*, *Bacillus pumilus*, *Bacillus subtilis*	For 10 weeks (8 weeks between August and September 2017 and 2 weeks in February 2018), weekly	Liquid soap detergent, pine liquid disinfectant, and ammonia (NH_3_)
La Fauci, 2015 [22]	5/2013–7/2013, Italy	Prospective, comparative interventional	Thoracic and vascular surgical wards	Wash basin, floor, desk, bed, bedside table, and door handle	*Bacillus megaterium*, *Bacillus pumilus*, *Bacillus subtilis* (30 × 10^6^ CFU/mL) with ionic surfactants (0.6%), anionic surfactants (0.8%), and amylases (0.02%)	For 3 months (6 May–30 July)	Chemical-based solutions
Leistner, 2023 [27]	6/2017–8/2018, Germany	Cluster-randomized, controlled, crossover trial	18 wards (not ICUs, 10 surgical, 8 medical) in a university hospital	Floor, door handle, washbasin, shower cubicle, and toilet surface	*Bacillus subtilis*, *Bacillus megaterium*, *Bacillus licheniformis*, *Bacillus pumilus*, *Bacillus amyloliquefaciens* (5 × 10^7^ CFU/mL, with a total concentration of 1%)	For 4 months (1 month wash-in period before the PBCS application)	For 4 months, consecutive applications of disinfectant (2-phenoxyethanol [10%], 3-aminopropyldodecylamine [8%], benzalkonium chloride [7.5%]); soap-based solutions used as a reference (non-ionic surfactants, anionic surfactants, and fragrances)
Soffritti, 2022 [28]	NR, Italy	Pre–post interventional	Emergency rooms of a “Maternal and Child Health Institute”	Floor, bed footboard, and sink	*Bacillus megaterium*, *Bacillus pumilus*, *Bacillus subtilis*	For 2 months	Pre-intervention: chemical sanitation; also, 0.5% NaOCl was permitted for application in confirmed cases of COVID-19
Tarricone, 2020 [29]	1/2016–6/2017, Italy	Pre–post interventional	5 public hospitals (plus a control hospital)	NR	10^7^ probiotics/mL, 1:100 dilution	Daily for 6 months	Pre-intervention: conventional cleaning solution (NaOCl 0.1%) daily for 6 months
Vandini, 2014 [31]	NR, Belgium, Italy	Pre–post interventional	Three hospitals (1 in Belgium, 2 in Italy)	Floor, door, shower, window sill, toilet, sink (made of stone, wood, plastic, glass, metal)	*Bacillus megaterium*, *Bacillus pumilus*, *Bacillus subtilis* (5 × 10^7^ CFU/mL)	For 6 weeks in 1 Italian hospital and for 24 weeks in 1 Italian and 1 Belgian hospitals	Chemical detergent (in the Belgian hospital), chlorine-based detergent (in Italian hospitals) for 6 weeks in 1 Italian hospital and for 24 weeks in 1 Italian and 1 Belgian hospitals
Vandini, 2014 [30]	3/2011–8/2011, Italy	Prospective, comparative interventional	One university hospital	Corridor, floor, sink	*Bacillus megaterium*, *Bacillus pumilus*, *Bacillus subtilis* (30 × 10^6^ CFU/mL) with ionic surfactants (0.6%), anionic surfactants (0.8%), and amylases (0.02%)	For 4 months	Chlorine-based solution (0.65% NaOCl, 0.02% surfactants)

Abbreviations: CFU, colony forming unit; ICU, intensive care unit; NR, not reported; PBCS, probiotic-based cleaning solution; Notes: ^a^ There was a two-month interval between the application of the probiotic-based cleaning solution and the control solution in two hospitals, and a six-month interval in three hospitals. ^b^ In February 2018, additional data were collected for the evaluation of HAIs.

**Table 2:** Outcomes of the use of probiotic-based cleaning solutions in clinical settings.

Author, Year [Ref #]	Presence of Pathogens in PBCS vs. Control	Presence of *Bacillus* spp. in PBCS vs. Control	Decrease in Resistance Genes	Emergence of HAIs in PBCS vs. Control (*n*/N [%])	Reduction in Pathogen CFUs (%)
Afinogenova, 2018 [14]	On day 30, no growth of pathogens (Enterobacterales, *E. faecium*, *Staphylococcus* spp.) was observed vs. 10^2^ CFU Enterobacterales and 10^3^ CFU *Staphylococcus* spp.	NA	NA	NA	NA
Al-Marzooq, 2018 [15]	In the spittoon area, heavy growth was reported for Gram-negative rods and *Streptococcus* spp.	NA	NA	NA	After PBCS application: *Staphylococcus* sp., 6.3–87.7; Gram-negative rods, 18.9–84.2; *Streptococcus* sp., 39.4–100
Caselli, 2016 [17,19]	Up to 98% CFU/m^2^ decrease in pathogen number	Four months after daily PBCS cleaning, *Bacillus* quota: 66.0 ± 5.5% vs. 6.7 ± 3.1%	83/84 (99%) pathogen genes decreased; no acquired resistant genes in *Bacillus* strains	6/159 (4) in PBCS (no data for control)	NA
Caselli, 2018 [18], Caselli, 2019 [16]	Median CFU/m^2^ (range ± SD): 4632 (842 ± 12,632) vs. 22,737 (17,053 ± 60,632), *p* < 0.001	*Bacillus* spp. quota, median (range): 69.8 (39.9–86.8) vs. 0 (0–30), *p* < 0.001	NA	Cumulative incidence: 128/5531 (2.3) vs. 283/5930 (4.8), range: 1.3–3.7%, *p* < 0.001; incidence rate ratio (95% CI): 0.45 (0.36–0.45) ^a^	Mean (range): 83 (70–96.3) 79.6
D’Accolti, 2023 [20]	After 2 weeks of daily PBCS application, median CFU/m^2^ (range), hospital 1 rooms: 3368 (0–87,579) vs. 7158 (0–91,789); hospital 1 bathrooms: 13,264 (0–124,211) vs. 20,211 (421–275,368); hospital 2 rooms: 3158 (0–114,526) vs. 8790 (0–158,900); hospital 2 bathrooms: 6948 vs. 16,420 (0–102,316). After 4 weeks, median CFU/m^2^ (range), hospital 1 rooms: 2947 (0–60,211) vs. 7158 (0–91,789), *p* < 0.05; hospital 1 bathrooms: 9895 (0–66,105) vs. 20,211 (421–275,368), *p* < 0.05 ^b^	Significant increase of *Bacillus* spp. after PBCS solution: more than 50,000 CFU/m^2^ in hospital 1, up to 30,000 CFU/m^2^ in hospital 2 (exact data not reported)	After 4 weeks of daily PBCS application, hospital 1: decrease in all resistance genes; hospital 2: decrease in some genes (*ermB*, *tetB*, OXA-23 group, OXA-51 group, *spa*) ^c^	NA	NA
Klassert, 2022 [24]	Decreased intrinsic microbiota in PBCS vs. other cleaning methods (disinfectants and detergents) in all surfaces (floor, door handle, and sink), for the sink: median 16S rRNA copies (IQR), PBCS vs. traditional disinfection: 138.3 (24.38–379.5) vs. 1343 (330.9–9479), *p* < 0.05	NA	Mean ± SD, antimicrobial resistance genes/sample, 0.095 ± 0.067 (PBCS) vs. 0.127 ± 0.037 (detergent) vs. 0.386 ± 0.116 (disinfectant), *p* < 0.01 ^d^	NA	NA
Kleintjes, 2019 [25]; 2020 [26]	24 vs. 12 pathogens, 4 pathogens had an unknown CFU count in the PBCS group; 1–10 CFU(s): 10/20 (50.0) vs. 6/12 (50.0); 11–100 CFUs: 8/20 (40.0) vs. 5/12 (41.7); >100 CFUs: 2/20 (10.0) vs. 1/12 (8.3)	NA	NA	8/2017–9/2017: 18/64 (28.0) vs. 149/264 (56.4); 2/2018: 4 new HAIs per patient are reported; monthly average difference 58.9% (lower in PBCS); *p* < 0.005	NA
Fauci, 2015 [22]	*E. faecalis* and *C. albicans*: complete elimination after 24 or 48 h of PBCS use; *A. baumannii*, *K. pneumoniae*: elimination in the first two months, but in the third month, no significant reduction of bacterial count	NA	NA	NA	NA
Leistner, 2023 [27]	Overall infection caused by MDR pathogens: 0.862 (0.434–1.710); *p* = 0.6757 vs. 0.919 (0.468–1.800); *p* = 0.81	NA	NA	IRR (95% CI): 0.955 (0.692–1.315); *p* = 0.84 vs. 0.953 (0.692–1.313); *p* = 0.83	NA
Soffritti, 2022 [28]	Before PBCS application, median CFU/m^2^ (95% CI): 26,315 (19,155–52,334); after 2 weeks, median CFU/m^2^ (95% CI): 6365 (4555–10,201); after 5 weeks, median CFU/m^2^: 5684; after 9 weeks, median CFU/m^2^: 41,461 ^e^	Before PBCS application, median CFU/m^2^: 991; after 2 weeks: 15,418; after 5 weeks: 17,447; after 9 weeks: 13,028	NA	NA	NA
Tarricone, 2020 [29]	NA	NA	NA	100/106 (94.3) vs. 191/203 (94.1); cumulative HAI incidence: 2.4% vs. 4.6%, OR (95% CI): 0.47 (0.37–0.60); *p* < 0.001 ^f^	NA
Vandini, 2014 [31]	Mean (95% CI) CFU/m^2^: coliforms: San Giorgio hospital (after 24 weeks) 125 (37–212), *p* = 0.002; Sant’Anna hospital (after 6 weeks) 764 (340–1188), *p* < 0.001; AZ Lokeren hospital (after 24 weeks) 3560 (3273–3846), *p* < 0.001. *S. aureus*: San Giorgio hospital (after 24 weeks) 286 (87–485), *p* < 0.001; Sant’Anna hospital (after 6 weeks) 5724 (4139–7309), *p* < 0.001; AZ Lokeren hospital (after 24 weeks) 627 (395–858), *p* < 0.001. *C. albicans*: San Giorgio hospital (after 24 weeks) 78 (0–162), *p* < 0.001; Sant’Anna hospital (after 6 weeks) 729 (365–1093), *p* = 0.001. *C. difficile*: AZ Lokeren hospital (after 24 weeks) 108 (40–177), *p* = 0.004 ^g^	NA	NA	NA	NA
Vandini, 2014 [30]	30 min after application (CFU/100 m^2^): *S. aureus*: 49.2 vs. 58.86 (*p* = 0.014), Coliforms 12.02 vs. 5.00 (*p* = 0.001), *Pseudomonas* spp.: 5.53 vs. 1.80 (*p* = 0.005), *Candida* spp.: 4.78 vs. 6.08 (*p* = 0.666); 6.5 h after application: *S. aureus*: 110.96 vs. 81.65 (*p* = 0.001), Coliforms 23.05 vs. 3.67 (*p* = 0.001), *Pseudomonas* spp.: 9.17 vs. 0.76 (*p* = 0.001), *Candida* spp.: 15.02 vs. 4.26 (*p* = 0.001)	NA	NA	NA	NA

Abbreviations: CFUs, colony-forming units; HAI, healthcare-associated infection; IRR, incidence rate ratio (incidence calculated as incidence per 100 patients/incidence density per 1000 exposure days); MDR, multidrug-resistant; NA, not available; PBCS, probiotic-based cleaning solution. Notes: ^a^ Cumulative incidence (HAI/total enrolled patients), incidence rate: incidence per 1000 patient-days. Also, length of stay in probiotic-based solution versus control (mean ± SD): 10.5 ± 6.7 vs. 9.7 ± 7.6. ^b^ Detailed data for hospital 1 were available after 4 weeks of evaluation. ^c^ In hospital #2 (H2), there was an increase in some genes (*msrA*, *oprj*, and *oprm*). Data were provided as figures, and thus no specific numbers were reported. ^d^ Antimicrobial resistance genes identified in all surfaces included *mecA*, *bla*_VIM_, *bla*_NDM_, and *bla*_OXA-48_. After application of PBCS, in 7/9 rooms, no antimicrobial resistance genes were detected. ^e^ After 9 weeks, NaOCl solutions were applied almost daily because of an increase in COVID-19 cases; thus, the action of PBCS was inactivated. ^f^ Cumulative HAI incidence was defined as: number of patients with HAI/total n of enrolled patients. ^g^ The *Clostridium difficile* isolates were monitored only in the AZ Lokeren hospital and the *Candida albicans* only in the San Giorgio and Sant’Anna hospitals.

**Table 3:** Characteristics and outcomes of the included studies that used probiotic-based sanitation cleaning systems for experimental (non-clinical) surfaces.

Author, Year [Ref #]	Setting	Tested Surface	Design and Duration of Probiotic Cleaning	PBCS Strain	Design, Duration (Solution) of Control	Presence of Pathogens in PBCS vs. Control	Presence of *Bacillus* spp. in PBCS vs. Control
D’Accolti, 2021 [21]	Suspension and surface tests	For the suspension tests, the “UNI EN 14476:2019” standard procedure was used; for the surface tests, stainless-steel sterile disks were used	According to the European standard procedure “UNI EN 16777:2019”, the antiviral activity of 100 μL of 1:10, 1:50, and 1:100 dilutions of PBCS was evaluated in suspension and surface tests	*Bacillus megaterium*, *Bacillus pumilus*, *Bacillus subtilis* (10^7^ CFU spores/mL)	NA	24 h after PBCS application: all tested viruses were eliminated with a mean of 0.1 virus titer (log_10_ TCID_50_/mL) in all PBCS dilutions (1:10, 1:50, and 1:100) in both suspension (MVA, HSV-1, hCoV-229E, human beta-coronavirus SARS-CoV-2, human H_3_N_2_, avian H_10_N_1_, swine H_1_N_2_) and surface (MVA and hCoV-229E) tests	NA
Hu, 2022 [23]	Simulation	Stainless-steel surface simulation of a high-touch surface in a healthcare environment	Probiotic cleaner in ambient and humid conditions	NA	Cleaning solution without probiotics	*A. baumannii*: maximum 8.75 log_10_ reduction compared to cleaning solutions without probiotics; *K. pneumoniae*: maximum 7.42 log_10_ reduction compared to cleaning solutions without probiotics	Gradually dominating with *Bacillus* spp. due to the elimination of pathogens
Stone, 2020 [11]	Surface tests on blocks with desiccated *E. coli* and *S. aureus*	Ceramic, linoleum, and stainless steel (placed indoors and outdoors)	Probotic cleaner (undiluted) twice a week for 8 months	*Bacillus* spores (8.6 × 10^7^ CFU spores/mL)	Plain soap (saponified vegetable extract, essential oils, natural gum), or disinfectant (3.5% *m*/*v* NaOCl) twice a week for 8 months	PBCS and plain soap both limited the survival of *S. aureus* and *E. coli* compared to disinfectant and tap water, on all surfaces, both indoors and outdoors	NA

Abbreviations: *A. baumannii*, *Acinetobacter baumannii*; *E. coli*, *Escherichia coli*; HSV-1, herpes simplex virus type 1; *K. pneumoniae*, *Klebsiella pneumoniae*; MVA, modified Vaccinia virus Ankara; NA, not available; PBCS, probiotic-based cleaning solution; *S. aureus*, *Staphylococcus aureus*; TCID_50_, 50% tissue culture infectious dose.

**Table 4:** Effect of probiotic-based cleaning solutions on antimicrobial resistance in clinical settings.

Author, Year (Isolates) [Ref #]	Studied Isolate	Antibiotic	Antimicrobial Resistance (*n*/N%)	Antimicrobial Resistance (*n*/N%)	*p*-Value
			PBCS	Control	
Al Marzooq, 2018 [15]	*S. aureus* (50 strains isolated from different surfaces were tested)	Ciprofloxacin	6/25 (24)	4/25 (16)	0.73
		Cotrimoxazole	5/25 (20)	8/25 (32)	0.52
		Cefoxitin	11/25 (44)	14/25 (56)	0.57
		Ceftriaxone	10/25 (40)	10/25 (40)	>0.99
		Cefpodoxime	17/25 (68)	19/25 (76)	0.75
		Cefepime	10/25 (40)	6/25 (24)	0.36
		Meropenem	7/25 (28)	4/25 (16)	0.50
		Gentamycin	0/25 (0)	1/25 (4)	>0.99
Al Marzooq, 2018 [15]	Gram-negative rods (40 strains isolated from different surfaces were tested)	Ciprofloxacin	0/20 (0)	0/20 (0)	NA
		Cotrimoxazole	2/20 (10)	0/20 (0)	0.49
		Cefoxitin	14/20 (70)	10/20 (50)	0.20
		Ceftriaxone	7/20 (35)	5/20 (25)	0.73
		Cefpodoxime	18/20 (90)	19/20 (95)	>0.99
		Cefepime	5/20 (25)	12/20 (60)	0.025
		Meropenem	0/20 (0)	0/20 (0)	NA
		Gentamycin	0/20 (0)	0/20 (0)	NA
Caselli, 2019 [16]	*S. aureus*	Penicillin G	18/30 (60)	53/81 (65)	NR
		Ampicillin	20/30 (67)	58/81 (72)	
		Vancomycin	2/30 (7)	31/81 (38)	
		Oxacillin	18/30 (60)	50/81 (62)	
		Cefotaxime	22/30 (73)	61/81 (75)	
		Imipenem	16/30 (53)	42/81 (52)	

Abbreviations: NA, not available; NR, not reported; PBCS, probiotic-based cleaning solution.

## Data Availability

The data used in this study are available upon request.

## Supplementary Materials

The following supporting information can be downloaded at: https://www.mdpi.com/article/10.3390/biology14081043/s1, Table S1: Search strings used in each resource.

## Abbreviations

AIMS	Australian Incident Monitoring System
ARG	Antimicrobial resistance gene
BPR	Biocidal Products Regulation
CDC	Centers for Disease Control and Prevention
CFU	Colony forming unit
ECDC	European Centre for Disease Prevention and Control
ECHA	European Chemicals Agency
HAI	Healthcare-associated infection
HSV-1	Herpes simplex virus 1
MDR	Multidrug-resistant
MVA	Modified vaccinia virus Ankara
PBCS	Probiotic-based cleaning solution
PCR	Polymerase chain reaction
PDR	Pandrug-resistant
PRISMA	Preferred Reporting Items for Systematic Reviews and Meta-Analyses
SARS-CoV-2	Severe acute respiratory syndrome coronavirus 2
SD	Standard deviation
UTI	Urinary tract infection
UV	Ultraviolet
WHO	World Health Organization
XDR	Extensively drug-resistant
