# Supplementary material for: Probiotic-Based Cleaning Solutions: From Research Hypothesis to Infection Control Applications
Source: Biology (Basel). 2025 Aug 13;14(8):1043. doi: 10.3390/biology14081043 (PMC12383857; doi:10.3390/biology14081043)
Supplement: Supplementary file 1 [file biology-14-01043-s001.zip › biology-3789901-Supplementary.pdf]

Supplementary Table S1. Search strings used in each resource.

Resource	Search string	Coverage in years	Date of search	Results
Google Scholar [a]	probiotic AND (sanitation OR cleaning)	NA	3 June 2025	39,700
Web of Science	probiotic AND (sanitation OR cleaning)	1900 – 2025	25 May 2025	794
Scopus	probiotic AND (sanitation OR cleaning)	1788 – 2025	25 May 2025	369
PubMed	probiotic AND (sanitation OR cleaning)	1946 – 2025	25 May 2025	254

[a] In Google Scholar, the first 1,000 articles of the 39,700 results were accessible for evaluation.
